# Disrupted Causal Connectivity Anchored in the Posterior Cingulate Cortex in Amnestic Mild Cognitive Impairment

**DOI:** 10.3389/fneur.2017.00010

**Published:** 2017-01-24

**Authors:** Hong Yang, Chengwei Wang, Yumei Zhang, Liming Xia, Zhan Feng, Deqiang Li, Shunliang Xu, Haiyan Xie, Feng Chen, Yushu Shi, Jue Wang

**Affiliations:** ^1^Department of Radiology, The First Affiliated Hospital of College of Medicine, Zhejiang University, Hangzhou, China; ^2^Department of CT/MRI, The First Affiliated Hospital of the Medical College, Shihezi University, Shihezi, China; ^3^Department of Radiology, Tongji Hospital, Tongji Medical College, Huazhong University of Science and Technology, Wuhan, Hubei, China; ^4^Department of Neurology, The First Affiliated Hospital of College of Medicine, Zhejiang University, Hangzhou, China; ^5^Department of Psychiatry, The Fourth Affiliated Hospital Zhejiang University School of Medicine, Yiwu, China; ^6^Center for Cognition and Brain Disorders, Affiliated Hospital, Hangzhou Normal University, Hangzhou, China

**Keywords:** resting fMRI, amnestic mild cognitive impairment, Granger causality analysis, default mode network, effective connectivity

## Abstract

Amnestic mild cognitive impairment (aMCI) is a transitional stage between normal cognitive aging and Alzheimer’s disease. Previous studies have found that neuronal activity and functional connectivity impaired in many functional networks, especially in the default mode network (DMN), which is related to significantly impaired cognitive and memory functions in aMCI patients. However, few studies have focused on the effective connectivity of the DMN and its subsystems in aMCI patients. The posterior cingulate cortex (PCC) is considered a crucial region in connectivity of the DMN and its key subsystem. In this study, using the coefficient Granger causality analysis approach and using the PCC as the region of interest, we explored changes in the DMN and its subsystems in effective connectivity with other brain regions as well as in correlations among them in 16 aMCI patients and 15 age-matched cognitively normal elderly. Results showed decreased effective connectivity from PCC to whole brain in the left prefrontal cortex, the left medial temporal lobe (MTL), the left fusiform gyrus (FG), and the left cerebellar hemisphere, meanwhile, right temporal lobe showed increased effective connectivity from PCC to the whole brain in aMCI patients compared with normal control. In addition, compared with the normal controls, increased effective connectivity of the whole brain to the PCC in aMCI patients was found in the right thalamus, left medial temporal lobe, left FG, and left cerebellar hemisphere. Compared with the normal controls, no reduced effective connectivity was found in any brain regions from the whole brain to the PCC in aMCI patients. The reduced effective connectivity of the PCC to left MTL showed negative correlation trend with neuropsychological tests (Auditory Verbal Learning Test-immediate recall and clock drawing test) in aMCI patients. Our study shows that aMCI patients have abnormalities in effective connectivity within the PCC-centered DMN network and its posterior subsystems as well as in the cerebellar hemisphere and thalamus. Abnormal integration of networks may be related to cognitive and memory impairment and compensation mechanisms in aMCI patients.

## Introduction

Alzheimer’s disease (AD) is a common neurodegenerative disease that presents mainly with memory impairment and decline in cognitive function. Mild cognitive impairment (MCI) is a stage of intermediate cognitive decline between normal aging and dementia. Amnestic mild cognitive impairment (aMCI) patients have a 10–15% annual risk of transitioning to AD (1). However, the neuropathological mechanism of MCI is currently unclear.

Resting-state fMRI (rsfMRI) is a method of functional brain imaging that has been extensively used to evaluate regional neuronal interactions that occur when a subject is not performing any explicit tasks. Zhang et al. used a regional homogeneity (ReHo) method to study AD, MCI, and normal control groups and found that in AD and MCI patients, results indicated reduced ReHo values in the medial prefrontal cortex (mPFC), the bilateral posterior cingulate cortex (PCC)/cuneus, and the left inferior parietal lobe (IPL) as well as decreased memory and cognitive ability. The lower ReHo values in AD and MCI patients suggested that cognitive and memory impairment were correlated with decreased spontaneous brain activity. Furthermore, the MCI group showed increased ReHo values in the left IPL when compared with the control group, suggesting compensation for the impaired cognitive regions (2).

Recently, many aMCI researches have largely focused on the different functional networks. Converging rsfMRI findings suggested default mode network (DMN) as a potentially valuable biomarker for aMCI (3). Assessing resting-state functional connectivity in patients with AD and MCI revealed that compared with the normal group, AD patients demonstrated decreased DMN function in the cuneus and PCC, and the reduced DMN was correlated with impaired cognitive functions (4). Another rsfMRI research about patients with aMCI revealed that reduced DMN functional connectivity correlated with cognitive decline (5). Bai et al. found that aMCI patients had increased functional connections between the PCC and the PFC. It was consistent with a compensative mechanism due to impaired function. Meanwhile, abnormal functional connectivity between the PCC and the temporal cortex played an important role in cognitive impairment in aMCI patients (6). These studies suggested that the abnormal DMN functional connectivity correlated with impaired cognitive function and a compensative mechanism in aMCI patients.

Effective connectivity represents the direct or indirect causal effect of one brain region on another brain region (7, 8). Granger causality analysis (GCA) is a relatively data-driven effective connectivity analytical method. GCA analyzes the direction of information flow between brain areas using time series of information processing and can depict resting-state directional brain networks (8, 9). Yan et al. applied independent component analysis (ICA) combined with multivariate Granger causality analysis methods to evaluate inner effective connectivity of the resting DMN and found reduced effective connectivity in the medial temporal gyrus, HC, and fusiform gyrus (FG), as well as the cuneus (PreCN)/PCC in aMCI patients, indicating a significant correlation between these regions and cognitive functions such as auditory and verbal learning. They also found increased effective connectivity between the HC and the frontal cortex, reflecting conserved memory in aMCI patients with cognitive impairment and decreased DMN functional activities (10). Using correlation-purged Granger causality analysis and gray matter atrophy as a variable, to analyze changes in four effective connectivity networks in aMCI patients, the following networks were used: DMN, hippocampal cortical memory network (HCMN), dorsal attention network (DAN), and fronto-parietal control network (FPCN). Liang et al. found that aMCI patients showed increased or reduced effective connectivity compared with the normal group. Certain effective connectivity among the networks (e.g., DMN → FPCN, DMN → HCMN, DAN → FPCN) showed a correlation with impaired cognition and memory, suggesting abnormal network connections and compensation in aMCI patients (11). A recent rsfMRI study about aMCI patients, with combined group ICA and Bayesian network learning approach, revealed altered directed connectivity weights between DMN regions in the fronto-parietal, temporo-frontal, and temporo-parietal pathways. Those abnormal directed connectivity could be a reflection or cause of impairment in these corresponding functions and a compensatory mechanism that is active in aMCI (12). These studies suggested that the abnormal directed connectivity weights not only inner DMN but also between the networks that related to the impairment in these corresponding functions and a compensatory mechanism in aMCI patients.

The DMN is a network that shows changes in brain activity at the resting state and includes the anterior cingulate cortex (ACC), the mPFC, anterolateral prefrontal cortex (LPFC), inside and outside the inferior parietal (MILP), cuneus, PCC, and the MTL (13, 14). The DMN is divided into two subsystems (14). The anterior subsystem containing the anterior DMN, such as the ventral and dorsal mPFC, uses the ACC as a connective node and combines with the PC/PCC to participate in self-referential mental idealization (15). The posterior subsystem, which includes the cuneus/PCC, the bilateral IPL, and the medial temporal lobe, is mainly involved in episodic memory retrieval (16), uses the PCC as a key connective node of the DMN, and integrates processed information in the two subsystems (17, 18). As per our knowledge, few studies focus on the effective connectivity between PCC-center DMN and its subsystem in aMCI patients.

In this study, using the coefficient GCA method—an algorithm based on a regression coefficient (19), in which a signed regression coefficient is used to assess Granger effects, a positive value suggests an excitatory effect, whereas a negative value suggests an inhibitory effect or negative feedback (20), we described changes in the DMN (the PCC as the key connectivity node) and its subsystems, in effective connectivity with other brain regions, as well as in correlations among them. Combined with neuropsychological tests, we aimed to learn more about the neuropathological mechanisms of aMCI.

## Materials and Methods

### Study Subjects

This study was approved by the Ethics Committee of the First Affiliated Hospital of Zhejiang University School of Medicine, and all subjects signed informed consent. The aMCI group included 16 patients (8 males and 8 females) ranging from 49 to 89 years old with an average age of 67.18 ± 11.32 years. The main complaint of patients was memory impairment with an average disease progression of 1.25 ± 0.39 years when they visited the doctor’s office at the Neurology Outpatient Department at the First Affiliated Hospital of Zhejiang University School of Medicine. The control group included 15 healthy elderly individuals (8 males and 7 females) ranging from 48 to 81 years old with an average age of 65.06 ± 10.36 years. For all participants clinical examinations included interviews, in the case of patients also with informant as well as psychiatric, neurological, and physical examinations, structural MRI and routine laboratory blood tests. Patients were rated with a series of standardized diagnostic and severity instruments, including the mini–mental state examination (MMSE), the Clinical Dementia Rating Scale, the activities of daily living (ADL), and a complete neuropsychological assessment. All aMCI patients fulfilled the criteria by Petersen et al. (1) as follows: (1) main complaint of memory impairment for more than 6 months and confirmed by the subject and an informant, (2) impaired memory function for age and education documented by an Auditory Verbal Learning Test (AVLT)-delayed recall score that is less than or equal to 1.5 SD of age-adjusted and education-adjusted norms, (3) preserved general cognitive function evaluated by a MMSE score of 24 or higher, (4) intact ADL, through a history from the subject and informant, and (5) not demented. Healthy control group inclusion criteria were as follows: no family history of AD, MMSE score of 28 or higher, matched with aMCI patients by the gender, age, and education levels, and all subjects were right-handed. Exclusion criteria included patients complicated with stroke, mental illness, drug abuse, systemic disease, moderate to severe high blood pressure, and diabetes. Table 1 shows the detailed demographic data and clinical/cognitive characteristics of these participants.

**Table 1:** **Comparison of general data between aMCI patients and normal controls**.

	aMCI group	Control group	*p* Value
Age (years)	67.18 ± 11.32	65.06 ± 10.36	0.591
Gender	8/8	8/7	0.859
Education (years)	7.13 ± 3.86	7.40 ± 3.13	0.805
MMSE	25.06 ± 0.93	29.67 ± 0.62	<0.001
AVLT-immediate recall	5.42 ± 0.92	6.87 ± 0.87	<0.05
AVLT-delayed recall	3.13 ± 1.02	5.60 ± 0.63	<0.05
AVLT-recognition	6.50 ± 1.32	10.80 ± 1.32	<0.05
CDT	2.56 ± 0.51	3.87 ± 0.35	<0.05
ADL	21.5 ± 3.41	19.67 ± 2.47	0.096
HAMD	4.19 ± 1.72	3.6 ± 1.68	0.345
CDR	0.5	0	

*aMCI, amnestic mild cognitive impairment; MMSE, mini–mental state examination; AVLT, Auditory Verbal Learning Test; CDT, clock drawing test; ADL, activities of daily living; HAMD, Hamilton depression scale; CDR, Clinical Dementia Rating Scale. Values are means ± SD. The p value was obtained using a Pearson χ^2^ two-tailed test with continuity correction for n < 5*.

*The p value was obtained by a two-sample two-tailed t-test*.

### Instruments and Methods

Neuroimaging data were collected at the First Affiliated Hospital of the College of Medical Science, Zhejiang University using a 3.0-T GE Signa HDx scanner (GE Healthcare, Hangzhou, China) equipped with an eight-channel head coil. The patients were asked to keep their eyes closed and be awake, not to think of anything particular. Sponge pads were placed on both sides of the lower jaw to limit head motion. Conventional axial T2WI MRI was first scanned to exclude white matter lesions, cortical ischemia, or other lesions. Then configuration data were collected through a 3D fast field echo T1-weighted sequence (axial, TR/TE = 8.2/3.2 ms, TI = 100 ms, flip angle = 12°, FOV = 240 mm × 240 mm, matrix = 256 × 256, slice thickness = 1 mm, no gap, in-plane voxel size 1 mm × 1 mm). Finally, the rsfMRI scans were performed using a GRE-EPI sequence: TR/TE = 2,000/40 ms, flip angle 90°, FOV = 24 cm × 24 cm, matrix = 64 × 64, slice thickness = 5 mm, and 1 mm interslice. Each volume comprised of 22 axial slices, and 200 time points were collected, lasted for 6 min and 40 s. The scan orientation was parallel to the anterior and posterior commissure and anterior skull base.

### Data Processing and Statistical Analysis

DPARSFA (21) and SPM8^1^ software packages were used for MRI data pre-processing. To take into account the impact from the uniform field effect of the resonance apparatus, as well as participants not adapting to the scanning environment during the initial part of the experiment, fMRI data from the first 10 volumes were eliminated. The subsequent 190 volumes were subjected to slice timing to compensate for the time difference between different slices in each volume. A head movement correction was then done, and the subjects whose head motion exceeded 2 mm in translation or 2° in rotation in any direction were excluded from the further analysis. Functional data were registered to a high-resolution structural image and subjected to simulation transformation into Montreal Neurological Institute space, with a resolution of 3 mm × 3 mm × 3 mm. A Gaussian kernel, with a full width at half maximum of 6 mm, was then used to smooth the data, followed by bandpass filtering (0.01–0.08 Hz) to remove low-frequency linear drift and high-frequency noises caused by breathing and heartbeat. Finally, various nuisance covariates including the WM and CSF mean time series and the motion time series from Friston 24-parameter head motion model were regressed out.

### Granger Causality Analysis

**Figure 1:**
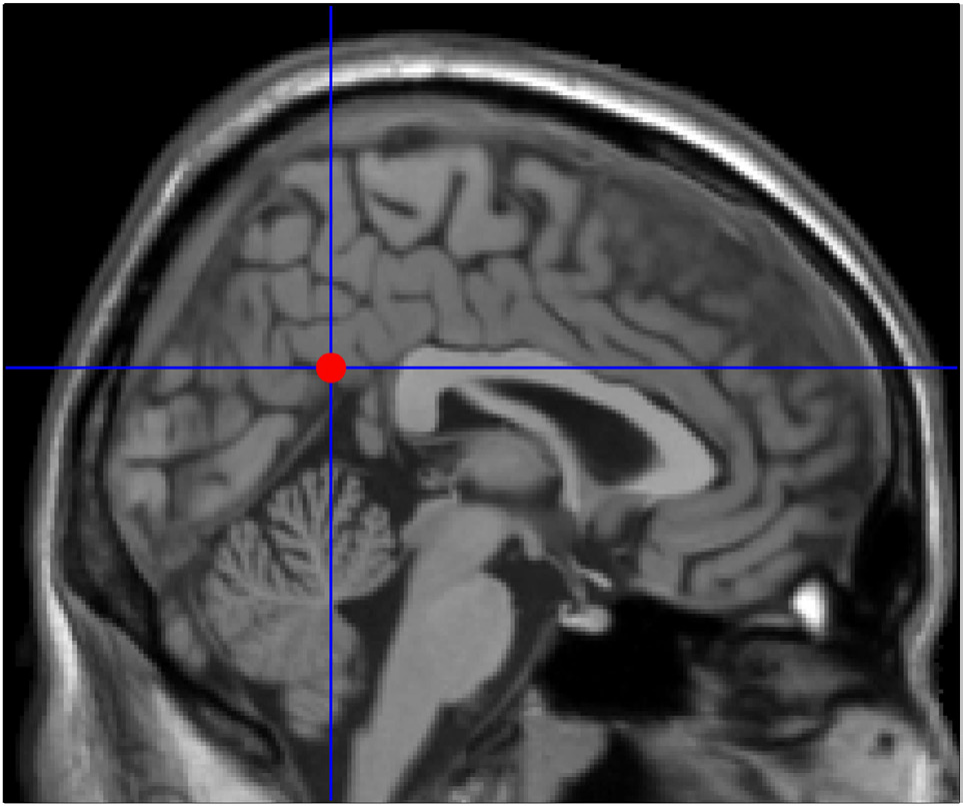
**Seed point in the posterior cingulate cortex (2, −51, 27) adapted from previously published articles on mild cognitive impairment neuroimaging**.

Granger causality analysis is a statistical hypothesis test for determining whether a time series is useful in forecasting another. The basic idea is to analyze two discrete time series, *X* and *Y*. If previous *X* and *Y* time series can more accurately predict the current *X* than the previous *X* time series alone, there is a GCA prediction relationship between *Y* and *X* (20, 22–24). Seed areas of the PCC were performed as previously described (25): coordinates: 2, −51, 27 with a radius of 5 mm (Figure 1). GCA was calculated using REST-GCA toolkit (26) implemented in the Resting-State fMRI Data Analysis Toolkit V1.8^2^ (27) and with the age as covariates. For estimating time-directed prediction between the fMRI time series of a seed region and the rest of the brain, a lag of one TR (2,000 ms) was used (28, 29). A signed-path coefficients algorithm was selected to obtain maps of PCC to whole brain and the whole brain to PCC. First, one sample *t*-tests were conducted. And to correct for multiple comparisons, we applied AlphaSim correction by using 3dClustSim in AFNI command lines^3^ within the whole brain mask, which is default mask from DPARSFA (voxel-height threshold *p* < 0.05, cluster = 236, corrected *p* < 0.05). Under the “causal effects mask,” the two groups were subjected to a two-sample *t*-test with the age, gender, and education as covariates, followed by another AlphaSim correction (voxel-height threshold *p* < 0.01, cluster = 44, and corrected *p* < 0.05). This process aims at improving the statistical significance of the final result. The analysis process for the causal effects from the whole brain to PCC was done as above. Then brain regions with abnormal results were used as regions of interest (ROIs), and the average ROI GCA values of each participant were calculated. Finally, the correlations between the mean GCA value of each ROI and the neuropsychological scores were calculated for the patient group.

## Results

### Demographic Data of the Study Participants

No statistically significant differences were found in gender composition, age, or educational levels between the aMCI group and healthy control group. However, statistically significant differences were found in MMSE as well as memory and clock drawing test (CDT) scores between the two groups (Table 1).

### Comparison of Effective Connectivity between aMCI and Healthy Control Groups

**Figure 2:**
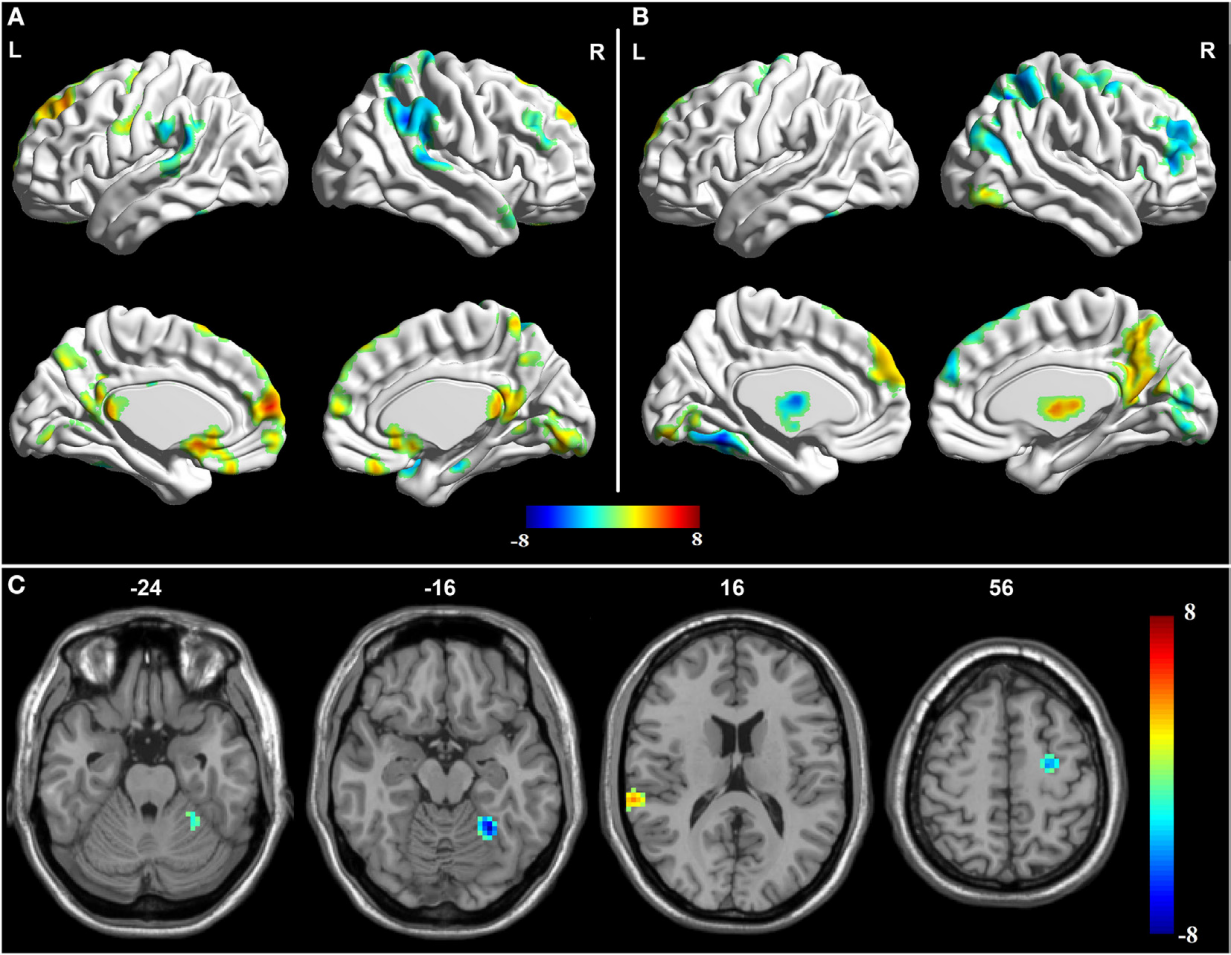
**Granger causality analysis for the posterior cingulate cortex (PCC) to whole brain**. **(A)** Brain regions showing significant causal effect with the PCC in controls. **(B)** Brain regions showing significant causal effect with the PCC in amnestic mild cognitive impairment (aMCI) patients. Warm and cold colors denote positive and negative causal effects (*p* < 0.05), respectively. **(C)** Brain regions showing group differences in causal effect from the PCC in a comparison of aMCI versus control. Blue areas show brain regions where aMCI patients had reduced causal effects than controls. The color bar represents *t*-values.

**Figure 3:**
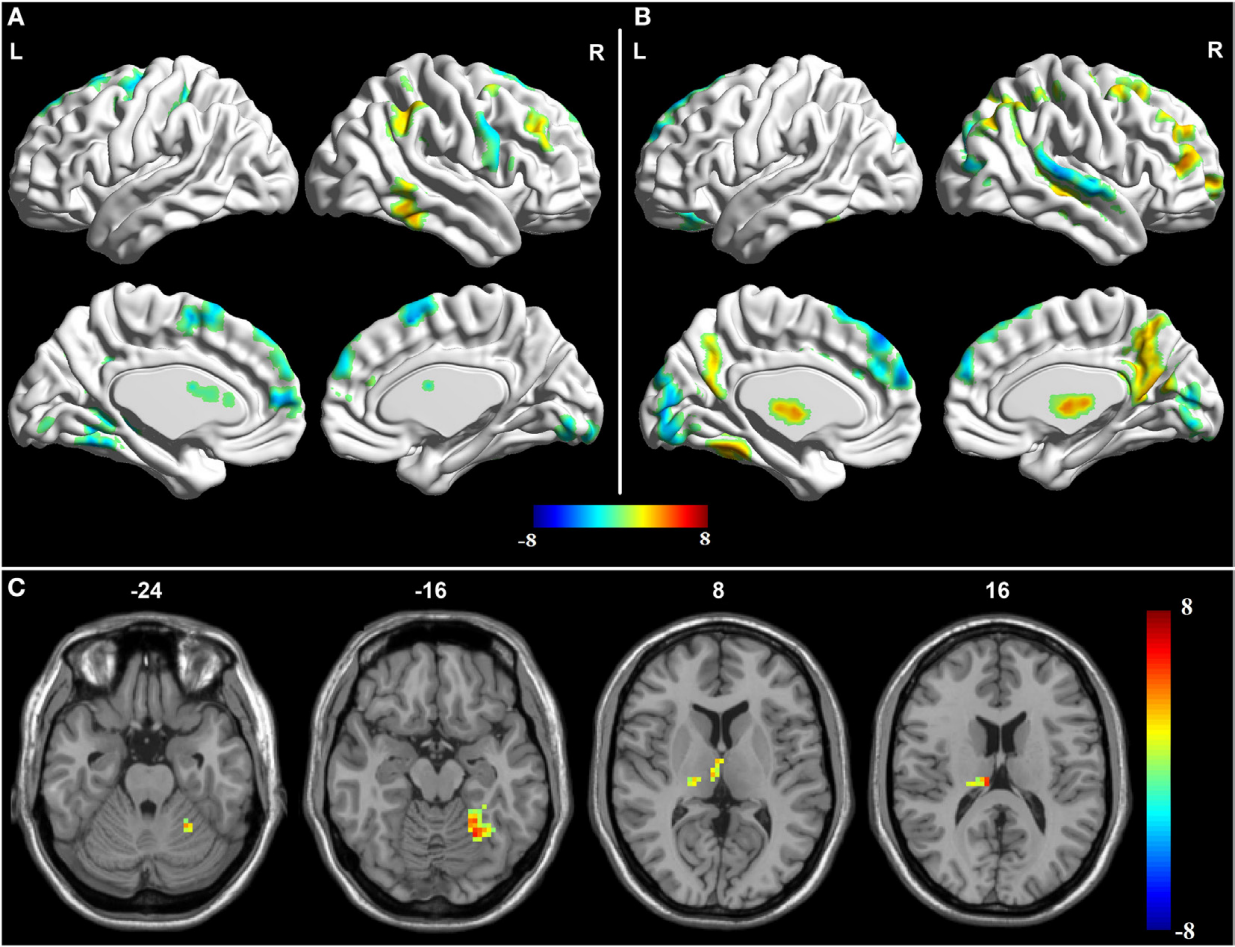
**Granger causality analysis for whole brain to posterior cingulate cortex (PCC)**. **(A)** Brain regions showing significant causal effect with the PCC in controls. **(B)** Brain regions showing significant causal effect with the PCC in amnestic mild cognitive impairment (aMCI) patients. Warm and cold colors denote positive and negative causal effects (*p* < 0.05), respectively. **(C)** Brain regions showing group differences in causal effect to the PCC in a comparison of aMCI versus control. Blue areas show brain regions where aMCI patients had reduced causal effects compared with controls, while red–yellow areas show brain regions where patients had increased causal effects compared with controls. The color bar represents *t*-values.

Compared with the normal group, the aMCI patients demonstrated decreased effective connectivity from the PCC to whole brain in the left PFC, the left MTL, the left FG, and the left cerebellar hemisphere, but right temporal lobe showed increased effective connectivity from the PCC to whole brain (Table 2 and Figure 2). In addition, compared with the normal group, the aMCI patients demonstrated increased effective connectivity from the whole brain to PCC in the right thalamus, left medial temporal lobe, left FG, and left cerebellar hemisphere, and no reduced effective connectivity was found in any brain regions (Table 2 and Figure 3).

**Table 2:** **Significant group differences in Granger causality analysis**.

Regions	MNI (*x*, *y*, *z*)	Cluster size (mm^3^)	*t* Value
**Effective connectivity from the PCC to whole brain**			
Left PFC	−24, −12, 57	86	−3.961
Left MTL	−25, −51, −12	80	−3.864
Left FG	−27, −46, −20	76	−4.278
Left cerebellar hemisphere	−28, −45, −24	83	−3.279
Right temporal lobe	66, −33, 12	93	4.459
**Effective connectivity from the whole brain to PCC**			
Right thalamus	14, −22, 15	84	2.945
Left MTL	−24, −47, −14	86	4.309
Left FG	−27, −48, −18	72	3.785
Left cerebellar hemisphere	−25, −52, −24	91	4.005

*MNI, Montreal Neurological Institute coordinates; PFC, prefrontal cortex; MTL, medial temporal lobe; FG, fusiform gyrus; PCC, posterior cingulate cortex*.

### Correlations between Abnormal Causal Interactions and Neuropsychological Scores in aMCI Patients

The brain regions with abnormal results including PCC to whole brain and whole brain to PCC were used as ROIs, and the average ROI GCA values of each participant were calculated. The correlations between the mean GCA value of each ROI and the neuropsychological scores were calculated for the patient group in SPSS toolkit.

**Figure 4:**
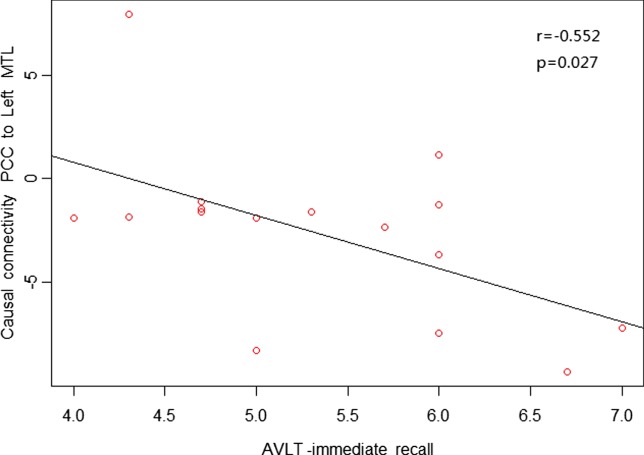
**Posterior cingulate cortex (PCC) to whole brain showed a negative correlation trend between causal connectivity and neuropsychological measures (including the AVLT-immediate recall) in the amnestic mild cognitive impairment group in scatter plots of these associations**.

**Figure 5:**
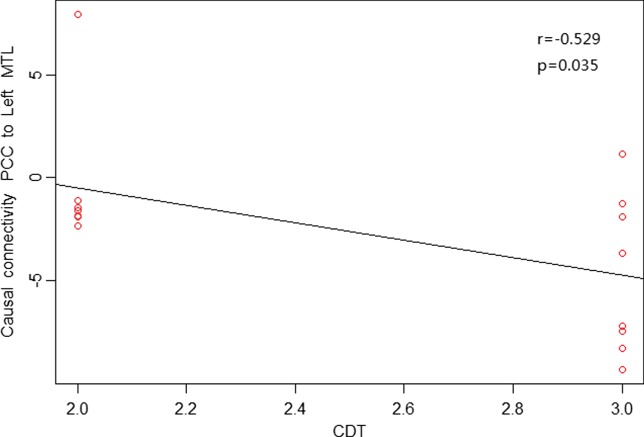
**Posterior cingulate cortex (PCC) to whole brain showed a negative correlation trend between causal connectivity and neuropsychological measures (including CDT) in the amnestic mild cognitive impairment group in scatter plots of these associations**.

As shown in Figures 4 and 5, in aMCI patients, effective connectivity from the PCC to left MTL showed a negative correlation with the AVLT-immediate recall (*r* = −0.552, *p* = 0.027); meanwhile, effective connectivity from the PCC to left MTL also showed a negative correlation (*r* = −0.529, *p* = 0.035) with the CDT. After removing the extreme value, effective connectivity from the PCC to left MTL showed a negative correlation trend with AVLT-immediate recall and CDT, the reason would be too small sample size. There was no linear correlation with the neuropsychological manifestations from the whole brain to PCC in aMCI patients.

## Discussion

Extensive research on network function in patients with MCI has focused on characteristic networks, such as the DMN (10) and the MTL network (30), and found that abnormal functional connectivity of the DMN and other networks was correlated with cognitive impairment and compensatory mechanisms in aMCI patients (5, 6, 10, 11, 31–33). In the current study, we used the PCC as connective nodes and applied the coefficient GCA to investigate the causality relationship between the PCC and the whole brain. The results showed that compared with the control group, aMCI patients showed (1) reduced effective connectivity from the PCC to left PFC, (2) reduced effective connectivity from the PCC to left MTL, and (3) increased effective connectivity from the PCC to right temporal lobe. A negative correlation trend was found between effective connectivity from the PCC to left MTL with the AVLT-immediate recall and CDT. These findings indicated impaired effective connectivity among DMN and its subsystems, using the PCC as a connectivity node, which is consistent with previous studies (10, 11, 31, 32). DMN might be a good candidate biomarker of AD prediction and early diagnosis. The increased effective connectivity from (1) the PCC to right temporal lobe, (2) the right thalamus to PCC, and (3) the left medial temporal lobe, the left FG, and the left cerebellum to PCC may indicate compensatory processes in the aMCI patients, which is also consistent with previous studies (34–37).

In the present study, reduced effective connectivity from the PCC to left PFC was found in aMCI patients compared with the normal group. The PFC is related to executive function, working memory, episodic memory, attention, decision-making, reasoning, and problem-solving functions. Previous studies of functional connectivity in aMCI patients also linked impaired cognitive function to decreased functional connectivity in the frontal lobe (3). A 3-year follow-up MCI study found more reduced functional connectivity from the superior and medical frontal gyrus to the PCC with the progression of disease (31, 32), indicating more severe frontal lobe dysfunction with progression of MCI.

Our study found reduced effective connectivity from the PCC to left MTL in patients with aMCI, suggesting impaired functional connectivity in the posterior subsystem (mainly the PCC and MTL). The MTL is considered the cornerstone of memory function in human brain and is closely related to the extraction of episodic memory. Das et al. further studied the network of anterior and posterior MTL in MCI patients and found impairment in both anterior and posterior networks; to some extent, the posterior MTL network was more prone to damage (30). Our results are partially consistent with their results, indicating damage in the posterior subsystem with the PCC as an important node in aMCI patients. Neuropsychological tests in this study also showed a negative correlation trend between effective connectivity from the PCC to left MTL with the AVLT-immediate recall (*r* = −0.552, *p* = 0.027) and CDT (*r* = −0.529, *p* = 0.035), suggesting that greater reduced effective connectivity leads to more severe memory and cognitive impairment. The increased effective connectivity from the PCC to right temporal lobe and left medial temporal lobe to PCC may reflect compensation for the posterior subsystems due to its impaired cognitive and memory function in aMCI patients. Previous studies have also confirmed that compared with the control group, aMCI patients had increased activity from the middle temporal gyrus and supramarginal gyrus to compensate for dysfunction in other brain regions (34).

The study found reduced effective connectivity from the PCC to left FG, but increased effective connectivity from the left FG to PCC. Early studies suggested that the FG is an important area of visual cognition such as facial recognition. Bokde et al. reported that MCI patients had extensive changes in effective connectivity of the FG in a face-matching task (38). The FG can relay information to the parahippocampal cortex and limbic cortex, and it plays a role (39) in memory processes *via* the entorhinal cortex and hippocampus connection. The study found reduced effective connectivity from the PCC to left FG, consistent with preliminary findings of reduced connectivity from the PCC to the right FG (6), indicating impaired local memory function in patients with aMCI. Increased effective connectivity from the left FG to PCC suggested that the left FG may be prompted to recorrect memory function and compensate. Cai et al. found increased connectivity from the left fusiform to the cuneus, which, together with the PCC, is part of the DMN (36). Our study was consistent with previous studies.

In addition, increased effective connectivity from the right thalamus to PCC was found in aMCI patients. Although not part of the DMN network, the thalamus has extensive connectivity with the cerebral cortex and subcortical structures and thus is an important center for subcortical information transmission and processing (40). In recent years, cross-sectional studies of the thalamus found abnormal functional connectivity between the thalamus and default network connections in some brain regions in AD and MCI patients, and this abnormality was closely related to clinical cognitive impairment (31, 32, 41). A study by Jeon et al. found that the PFC is linked to the caudate nucleus and thalamus. The dorsolateral prefrontal loop passes information from the PFC *via* the CN, the pallidum/substantia nigra, and the thalamus, back to the PFC (35). This loop is known to be recruited in cognitive aspects including working memory, planning, rule-based learning, and sequence learning (42). This study found increased effective connectivity from the thalamus to PCC, which may reflect compensation of cognitive and memory impairment in aMCI patients.

Imaging studies showed that in addition to participation in movement and balance, the cerebellum is involved in many higher cognitive functions (43) as part of the cognitive network with the PFC and parietal lobes (44). The cortex–hypothalamus–cerebellar network is an important neural circuit (45). In this study, reduced effective connectivity from the PCC to the left cerebellum suggested cerebellum-associated cognitive impairment in aMCI patients. Furthermore, increased effective connectivity from the left cerebellum to PCC possibly reflected compensatory mechanisms of the cerebellum. Bai et al. revealed abnormal functional connectivity from the cerebellum to the hippocampus and many other brain regions in patients with AD and aMCI (37). Gloria et al. evaluated the resting-state brain network in patients with dementia (37) for cerebrum–cerebellum integrity of bidirectional correction and found that the effective connectivity coefficient of cerebellar function was reduced in the AD group and increased in the MCI group, indicating that the cerebellum takes part in a homeostatic mechanism that limits the progression of cognitive decline from MCI to AD (46).

It is noteworthy that in this study, the regions of increased functional connectivity in temporal lobe, thalamus, and cerebellum are involved in working memory or short-term memory, which are relatively preserved in early MCI (47, 48). Therefore, we speculate that MCI patients can partially compensate for impaired cognition through more effective use of relative retention of working memory or short-term memory. Similarly, clinical memory-related scores also showed that immediate recall, delayed recall, and long-delayed recognition were different (*p* < 0.05) in the aMCI group compared with the normal control group in our study. However, immediate recall showed a negative correlation trend between effective connectivity from the PCC to left MTL, suggesting that greater reduced effective connectivity leads to more severe immediate recall.

This study may have some inevitable limitations. First, the sample size is relatively small, resulting in unavoidable bias. Second, application of coefficient GCA in rsfMRI for data processing and low-frequency filtering as well as removal of whole brain average signals is controversial. No consolidated standard was formed in previous GCA studies. Thus, how this method affects the results needs further examination (49). The variability in the hemodynamic response function (HRF) can lead to spurious connectivity in fMRI study. A deconvolution technique is not applied in this study so that HRF variability affect to the results is unavoidable. Finally, TR in our rest-state fMRI study is 2 s with limited time resolution for GCA. While other techniques electroencephalography and magnetoencephalography (MEG) with high time resolution but MEG has a lower spatial resolution for deeper regions (50). With the development of MRI techniques, subsecond scan sequences of the whole brain will be obtained in rsfMRI studies, and this problem can be solved in the future.

## Conclusion

Using the coefficient GCA method, the differences of effective connectivity have been found in the PCC-centered DMN and its subsystems as well as in the cerebellar hemisphere and thalamus in aMCI patients. The differences in effective connectivity were related to cognitive and memory functional impairment and compensation for those dysfunctions. The aMCI patients may simultaneously partially compensate impaired function to reduce cognitive changes and prevent MCI to AD transition *via* different neural circuits.

## Author Contributions

HY is the corresponding author and first author for the manuscript and has participated in the data collection, data analysis, and writing. CW and YZ participated in the data collection, data analysis, and writing. ZF assisted in data collection and data analysis. DL, SX, FC, and YS assisted in data collection. LX, HX, and JW assisted with data analysis.

## Conflict of Interest Statement

The authors declare that the research was conducted in the absence of any commercial or financial relationships that could be construed as a potential conflict of interest.

## Footnotes

^1^http://www.fil.ion.ucl.ac.uk/spm.

^2^http://www.restfmri.net.

^3^https://afni.nimh.nih.gov/.
